# Interplay between policy and science regarding low-dose antimicrobial use in livestock

**DOI:** 10.3389/fmicb.2014.00086

**Published:** 2014-03-07

**Authors:** Amanda C. Sorensen, Robert S. Lawrence, Meghan F. Davis

**Affiliations:** ^1^Department of Environmental Health Sciences, Johns Hopkins Bloomberg School of Public HealthBaltimore, MD, USA; ^2^Center for a Livable Future, Environmental Health Sciences, Johns Hopkins Bloomberg School of Public HealthBaltimore, MD, USA

**Keywords:** antibiotic resistance, livestock, policy, funding, surveillance

Since the introduction of penicillin in the 1940s, antimicrobial resistance (AMR) has become an ever-increasing threat to human and animal health (Carlet et al., 2012; ITFAR, 2012). The low-dose antimicrobial use in food-producing animals for production purposes, i.e., disease prevention and growth promotion, has been documented to drive AMR, though the risk to human health from such uses has been a source of a heated debate among stakeholders including industry members, citizen advocacy groups, academics, and public health workers (Davis and Rutkow, 2012). In the United States (US), it remains common practice to use medically-important antibiotics^1^ for production purposes. Since the 2000s, the European Union (EU) has banned many low-dose antimicrobial uses. The crucial element to the debate has been whether scientific evidence supports similar action in the US. Industrial food animal production advocates, henceforth “industry,” claims that scientific evidence is currently lacking, while other stakeholders argue that a convincing and substantial body of evidence exists to support policy change.

The US and the EU have taken vastly different approaches to AMR surveillance and research, and these choices have led to a wide variance in the current policy climate regarding antimicrobial use and AMR. We argue that the EU has prioritized scientific research and surveillance efforts that target uses of antimicrobials in animals. The US has not invested deeply in either research funding or surveillance programs that include such uses. If policy shifts will occur in the US, changes in AMR surveillance and allocation of scientific research funding are required.

## Current US practices

The US National Antimicrobial Resistance Monitoring System (NARMS) is a joint effort of the US Department of Agriculture (USDA), the Food and Drug Administration (FDA), and the Centers for Disease Control and Prevention (CDC) for AMR surveillance. The three arms of NARMS—VetNet (USDA), PulseNet (CDC), and retail (FDA)—collect samples from animal carcasses at the time of slaughter, human food-borne infections, and retail meat samples, respectively. Techniques in each arm vary in regards to both isolates collected and laboratory definitions for resistance, making data comparison across branches challenging (US GAO, 2011). Collection of data on antimicrobial use and sales in food animals in the US is limited, though the FDA has made recent efforts to obtain more data. The Government Accountability Office, in a report published in 2011, commented on the limited scope of sampling techniques in the US:

This non-random sampling method means the NARMS data obtained … are not representative of food animals across the country and cannot be used for trend analysis because bacteria tested by NARMS are now collected at greater rates from slaughter plants that are not in compliance with food safety standards. According to FDA officials, due to this sampling method, the resulting data are skewed for NARMS purposes (US GAO, 2011).

The lack of harmonization within NARMS limits the interpretation of these data, with resulting impacts on evidence available to support science-driven policy.

Current NARMS funding is approximately $7.8 million, bringing the per-capita investment in this surveillance tool to $0.025 (US GAO, 2011). Lack of funding is routinely cited as the primary cause of limited government-led research and lack of advancement in surveillance techniques, though the lack of coordination within the current approach suggests that changes could be made to use existing budget funds more effectively (Pew Trust, 2008; US GAO, 2011; IDSA, 2012).

## Current european practices

The European approach to AMR surveillance, research, and policy development began with individual country efforts, with Denmark and Sweden as leaders. In recent years, the EU has begun to compile data from numerous countries through the functions of the European Centers for Disease Control, European Antimicrobial Susceptibility Surveillance in Animals Center, and the European Animal Health Study. In general, European efforts have been more systematic than US efforts, including sampling of healthy and diseased animals and people, monitoring antimicrobial usage patterns through novel techniques such as geomapping, and using veterinarians to obtain samples from a wide variety of regions and animals, including pets (DANMAP, 2011; Davis and Rutkow, 2012; IDSA, 2012; ITFAR, 2012; de Jong et al., 2013).

Selective bans on antimicrobials for growth promotion were instituted in the 1990s, and a complete ban of drugs for non-therapeutic uses was established in Denmark and the EU in 2000 and 2006, respectively (DANMAP, 2011; US GAO, 2011). Research is prioritized and is typically led by industry rather than government; this approach may reflect differences in cultural or social norms between the EU and US. Indeed, industry participation in such programs has been largely voluntary, and the additional cost is thus often internalized to industry rather than externalized to the public through government expenditures.

## Comparison of approaches

According to the 2011 GAO audit of NARMS, policy in the EU, “… has been built around the precautionary principle, which states that where there are threats of serious or irreversible damage, lack of scientific certainty should not postpone cost-effective measures to reduce risks to humans” (US GAO, 2011). In contrast to the US approach of risk assessment, the EU method is buoyed by consumer concern and voluntary industry measures. At a recent meeting, the Infectious Disease Society of America (IDSA) released a statement comparing current US and EU efforts:

Even given the value [National Health Safety Network], [Emerging Infections Program], Prevention Epi-centers, NARMS, and [Multidrug Resistant Repository and Surveillance Network] provide, IDSA remains deeply concerned about the lack of detailed, publicly available data on both resistance trends and human antimicrobial use in humans, food animals and other areas of agriculture and food production in the United States. The U.S. is far behind other countries in collecting and benefiting from data on antibiotic consumption and resistance (IDSA, 2012).

In the same vein, the 2011 GAO report noted that CDC did not routinely publish data on AMR patterns in pathogens associated with outbreaks of foodborne illness (US GAO, 2011). Such statements indicate the low political priority assigned to AMR surveillance and program evaluation in the US. Evaluation is a vital part of any system, and as the 2011 GAO report indicated, NARMS, FDA, USDA, and CDC have conducted few evaluations. Without regular evaluation, programs and policies that are neither cost-effective nor impactful can waste money and prevent new and more efficacious systems from being put in place.

DANMAP was established in 1995 with 2.4 million kroner ($0.3 million) and in 2000 further supported with 2.6 million kroner resulting in a total of 5 million kroner ($0.7 million) of initial investment across that time period.^2^ This program was one of the pioneers in European surveillance efforts, predating EU-wide efforts (Davis and Rutkow, 2012). Currently, DANMAP is not funded by earmarked money but is a part of the general tasks of Statens Serum Institut and Danish Technological University (DTU-Food) financed by Danish Ministry of Health and the Danish Ministry of Food, Agriculture, and Fisheries. Although the budget for DANMAP changes relative to general fund availability and is subject to annual decreases, the budget from the Ministry of Food, Agriculture and Fisheries alone for all food research (not just application to AMR) was 246 million kroner ($44.8 million), or a per-capita investment of $8 for 2014^3^. Funding across multiple federal agencies places US food research investment at an estimated $1863 million, or a per-capita investment of less than $6 for 2013^4^. Given similar per-capita budgets, the food safety and DANMAP programs have more effectively influenced AMR policy in Denmark than parallel programs have in the US. This example suggests that the choice of where and how to apply funding may be more important than total expenditure in the generation of scientific evidence to support policy decisions.

The role of industry in the US is complex, and the 2008 report of the Pew Commission on Industrial Farm Animal Production noted, “We found significant influence by the industry at every turn: in academic research, agriculture policy development, government regulation, and enforcement” (Pew Trust, 2008). Industry itself is not held accountable for ensuring public health, nor is it charged with contributing peer-reviewed research to support or refute its current or preferred practices, yet it remains able to exercise tremendous influence in government and academia, especially among land grant universities where industry funding has largely replaced public research funding (Pew Trust, 2008).

Hence, policy success, defined as both action by agencies and reductions in AMR rates, in the EU has been driven in part by strategic allocation of funding support, adherence to the precautionary principle, involvement of industry, and periodic mandatory participation of EU members in region-wide data collection efforts (DANMAP, 2011; IDSA, 2012; Silley et al., 2012). In contrast the US has not seen the same returns relative to funding efforts, has not applied the precautionary principle in formulating policy, has not required participation of producers or benefited from voluntary support by industry, and has conducted limited and flawed surveillance for antimicrobial use in livestock and AMR.

## Recommendations

In an era of resource constraints for government agencies, we do not expect AMR surveillance efforts in the US to experience significant budget increases. The EU approach, including the specific example of Denmark, offers lessons for change in the US. We propose that efforts be made to provide minimal increases in funding to obtain more representative and comparable data, and to analyze data currently being collected in a more effective way. For example, NARMS data in the US are available online, but manipulation of the data is not feasible (Silley et al., 2012). Allowing independent researchers and their students to use the NARMS data for analysis may lead to more rapid reports that can be used for comparison with data from other countries, while supplying a no-cost source of analysis and enhancing educational opportunities. In particular, provision of data at the sample level, allowing for analysis of cross-resistance patterns, would inform both scientific and policy efforts to understand and combat the rise of AMR pathogens.

Additionally, a shift in industry's role toward a more harmonized partnership with government and other stakeholders would assist national efforts to address the problem of AMR. Voluntary FDA guidance to phase-in veterinary oversight and phase-out growth promotion uses of antimicrobials in livestock is a first step toward this goal (FDA, 2013). Following the EU example for industry involvement in research efforts would ultimately offer a solution to meet the needs for representative sampling within a country as large and diverse as the US. While industry may be faced with higher costs, all stakeholders would benefit from improvements in data collection—and hence data interpretation—in driving evidence-based policy change.

Ultimately, changes need to occur within the US to encourage industry accountability, research efforts, and government investment in adequate surveillance systems. Some improvements within the US surveillance system, such as reporting of data already collected, would require minimal funding adjustments. AMR is a pressing problem that threatens the health and well-being of humans and animals, with impacts through the food system. Fundamental shifts in the governmental and industry approaches, including structural changes to enhance data collection and dissemination, are urgently needed to generate science-based policies and then understand the impact of the policies on AMR.

## Footnotes

^1^Based on definitions for critically-important antimicrobials used by the Food and Agriculture Organization of the United Nations, World Health Organization, and World Organization for Animal Health (OIE), and definitions of medically-important antibiotics in the Preservation of Antibiotics for Medical Treatment Act, first introduced to the U.S. Congress in 1999.

^2^Based on a year 2000 exchange rate of 7.3855kr/US dollar. With a year 2000 Danish population of 4.8 million, the per-capita investment was roughly $0.14. We thank Robert Skov for his assistance in obtaining this DANMAP funding information.

^3^Based on a December 1, 2013 exchange rate of 5.489810kr/US dollar. With a year 2013 Danish population of 5.6 million, the per-capita investment was $8.

^4^Based on the sum of food safety budget estimates of $42 million (NIFA), $108 million (ARS), $1 million (ERS), $1425 (FDA), and $287 (NIH), for a total of $1863 million (Ohlhorst et al., 2013). With a year 2013 US population of 320.6 million, the per-capita investment was $5.8.
